# Prevalence of temporomandibular disorders and its association with malocclusion in children: A transversal study

**DOI:** 10.3389/fpubh.2022.860833

**Published:** 2022-09-09

**Authors:** Monica Macrì, Giovanna Murmura, Antonio Scarano, Felice Festa

**Affiliations:** Department of Innovative Technologies in Medicine and Dentistry, University “G. D'Annunzio” of Chieti- Pescara, Chieti, Italy

**Keywords:** TMD, TMJ, bruxism, teeth clenching, adolescent, children, developmental age

## Abstract

This study investigated the prevalence of temporomandibular disorders (TMDs) in a sample of children and adolescents and evaluated the correlation with occlusal variables. TMD signs and symptoms were recorded in 411 subjects (age range 7–15 years), divided into two groups: 214 subjects treated in Chieti (Italy) CG group and 197 in Murcia (Spain) MG group. Once the Angle dental class was identified, it was recorded if there were signs and symptoms of temporomandibular disorders (TMDs) and occlusal interferences. The percentages of signs and symptoms were compared to determine the differences among the groups for TMDs, bruxism, joint sounds, deviation during the opening, reduced opening/lateral/protrusive movements, malocclusions, and myofascial pain. There is no statistically significant difference between the two groups (χ^2^ = 1.057, *p* > 0.05). Subjects with Angle Class I (37.37%), deep bite (43.43%), and increased overjet (41.41%) showed a higher prevalence of TMD symptoms.

## Introduction

Temporomandibular disorders (TMDs) are a heterogeneous group of musculoskeletal and neuromuscular conditions which involve the temporomandibular joint complex. TMJ sounds, pain during mandibular function, limitation of mandibular movements, headache, and facial and neck pain are common signs and symptoms of TMDs (1). In childhood, such signs and symptoms are mild, while increasing slightly during adolescence in prevalence and severity (2–4). It is generally recognized that TMJ disorders have a multifactorial etiology. The literature suggests that TMD neuromuscular and mechanical, structural, and psychosocial factors are sources (5, 6). Besides, from several reviews, it emerges that the prevalence of TMD symptoms in patients with dentofacial deformities is higher than that of the general population (7, 8). A probable causal relationship between malocclusion and TMDs was supported for years and dental occlusion's role in predisposing and initiating temporomandibular disorders TMDs (9, 10). The occlusal paradigm for TMD has never been convincingly validated; however, the influence of occlusion as an etiological factor of TMDs is low (11, 12). Several studies have questioned the potential role of malocclusion in the onset of TMDs, concluding that there is no evidence to assume an essential part of dental occlusion in TMDs pathophysiology (13–16). After dental pain, TMDs are the most common cause of orofacial pain. Various studies have shown that TMD affects 10%−15% of the adult population, but only 5% require treatment (17). The highest incidence of TMD occurs from 20 to 40 years of age; in women, it is two times as high as in men (18).

Other age groups may also be affected by TMD, as the frequency is lower; therefore, studying TMD in different age groups, such as the elderly, children, and adolescents, is equally relevant (19, 20).

Bonjardim et al. (21) reported that TMD signs and symptoms were 34% in Brazilian children with primary dentition.

According to previous studies in European countries, TMD prevalence rates were 26.5% in Poland (22) and 28.21% among subjects aged 12–15 years and 22.58% among those aged 5–11 years in Italy, as reported by Tecco et al. (23). Slightly lower at the rate observed in China as described by Xie et al. (23, 24) who investigated a group of Chinese students from 1979 to 2017 and reported a 29.1% TMD prevalence and joint sounds (17.4%) as the most frequent sign. Higher prevalence rates appeared in the Middle East and South American countries: 34.7% in Iran (24), 46.8% in Riyadh (Saudi Arabia) (25), and 34.9% in Brazil (26). This discrepancy can be related to race, economy, war, and eating habits. Due to the different types and qualities of the analysis methods, the prevalence of TMD may vary (27). Epidemiological studies on TMD carried out in children apply the same methods as those used for adults. However, adjustments should be made, considering the different growth patterns of the masticatory system and the different levels of understanding and ability to discriminate against different situations in childhood (28).

This study carried out in the Department of Innovative Technologies in Medicine & Dentistry Oral Medical and Biotechnological Sciences Department of the “G. D'Annunzio” University of Chieti-Pescara aims to establish the prevalence of TMJ disorders in developmental age and to assess the relationship with malocclusions. TMD signs and symptoms were recorded in two groups of the same age. Subsequently, a comparison was made between the data collected at the Department of Innovative Technologies in Medicine & Dentistry of the “G. D'Annunzio” University and those at the “Clinica Universitaria Odontologìca” of Murcia (Spain).

## Materials and methods

Four hundred eleven patients were selected in this study (mean age 10.9 ± 2.1 years; age range 7–15years) divided into two groups; 214 (mean age 11 ± 1.9 years; age range 8–15years) from the University “G. D'Annunzio” Chieti-Pescara, Italy (CG group) and 197 (mean age 10.9 ± 2.3 years; age range 7–14 years) from “Clinica Universitaria Odontologìca,” Universidad de Murcia, Spain (MG group). All these patients were referred to Chieti and Murcia Clinic for orthodontic advice.

The following inclusion criteria were used for subject participation in the study: (1) 7–15 years of age and (2) patients who have come for their first orthodontic examination.

Patients were excluded if they had a history of polyarthritis, muscle spasms, neurological or psychiatric disorders, vascular diseases, genetic syndromes, cleft lip, palate abnormalities, and craniofacial syndromes.

The occlusal assessment was made for these variables: Angle malocclusion classification, the overjet, the overbite, the dental crowding, the presence of crossbite involving one or more teeth, the presence of deep or open bite, and the dental midline discrepancy. The patients were first classified dentally by evaluating the molar relationship considering the Angle norms. Class II included bilateral or unilateral mesial displacement of the upper first molar and canine at least half a cusp. Class III included patients with a bilateral or unilateral mesial displacement of the lower first molar and canine of at least half a cusp. To measure the incisal relationship, a horizontal line has been drawn on the facial surface of the mandibular incisor, using the incisal edge of the maxillary central incisor as a guide. The extent of horizontal and vertical overlap was measured with a ruler. Overjet values <3 mm were considered normal, and values ≥3 mm were considered increased; overbite was recorded as expected if the maxillary central incisors overlapped the mandibular central incisors crown for up to 4 mm; open bite was recorded when no overlap was seen between upper and lower incisors, namely, edge-to-edge relationship. The posterior crossbite was recorded when the buccal cusp of any of the maxillary premolars and molars occluded palatal to the buccal cusp of the antagonist mandibular teeth.

A clinical assessment for TMD signs and symptoms was performed according to the DC/TMD protocol (29) and the adaptation of the Axis I of the DC/TMD for use in children and adolescents (30). It was evaluated that myofascial pain in various body areas (head, face, jaw, neck, and shoulders) was measured with the VAS (31) (visual analog scale, which consisted of a graphic representation of the patient's face to assess the pain intensity from 0 to 10), TMJ sounds, sleep bruxism and awake bruxism, the deviation during the opening, the reduced opening, lateral, and protrusive movements. Parafunctional habits, such as onychophagy or atypical swallowing, were evaluated too. One specialist performed the examinations, previously instructed, using the DC/TMD Axis I by a reference examiner (FF) with expertise in TMJ disorders. Masticatory muscles were digitally palpated bilaterally using one finger (masseter, temporal, temporalis tendon, digastric anterior belly) according to DC/TMD protocol (29), functionally manipulated (lateral and medial pterygoids) according to Okeson (32), to assess muscle tenderness and pain (33). The palpation pressure, calibrated by an algometer, was 1 kg for 4–5 s to cause widespread or referred pain if present (masseter, temporal) (29, 30). A pressure of 0.5 kg was exerted to palpate supplemental muscles (i.e., digastric anterior belly, temporalis tendon) (29). As for functional manipulation (32). each muscle is contracted and then stretched. If a muscle is the source of pain, these two activities increase it. The pressure of 0.5 and 1 kg for 5 s was exerted for the palpation of the TMJ lateral pole and around the pole, respectively, and to determine the presence of joint sounds (click, crepitus, eminence click) during open, close, lateral, and protrusive movements (29, 30). The “Diagnostic Decision Trees” were used to evaluate pain-related TMD (myalgia, local myalgia, myofascial pain with spreading and with referral, arthralgia, headache) and intra-articular and degenerative joint disorders (i.e., disc displacement with or without reduction, with or without limited opening) (29).

A pain scale was used to assess the intensity of the pain (from 0 to 3). The pain scale has been illustrated to each patient to indicate the score exactly after each palpation recorded on both the right and left sides (0 = no pain/pressure only; 1 = mild pain; 2 = moderate pain; 3 = severe pain). In addition, regarding the VAS (31), the patient had to indicate the painful areas quantify the intensity (from 0 = no pain to 10 = maximum pain), and describe the pain characteristics and how/if it affects normal daily activities and if it is familiar to the pain that the patient may have experienced in the same region in the last 30 days according to DC/TMD protocol.

The opening pattern was evaluated by drawing a vertical line on the labial surface of the maxillary and mandibular reference incisor. Any deviation in the opening was recorded. The protocol used for the examination of jaw movements and the report of pain if present on movement was the same used in the adult version, but considering limited opening values, those with a lower threshold of 36 and 32 mm at 10 and 6 years of age, respectively, as reported in the study of Müller et al. (34) To evaluate joint sounds were asked to each patient if any TMJ sound was present with jaw movements or function in the last 30 days and describing it. Moreover, it was asked to open and close the mouth slowly three times, recording the sound as “click” (e.g., click, pop, snap) or “crepitus” (e.g., grating, grinding) if present in at least one of the three movements and detected with palpation. The same evaluation was performed for lateral and protrusive movements (29).

Signs of sleep bruxism and awake bruxism were distinguished between them. *Sleep bruxism* is a repetitive, rhythmic, or non-rhythmic, masticatory muscle activity during sleep that is not a movement or a sleep disorder in otherwise healthy individuals. *Awake bruxism* is a masticatory muscle activity during wakefulness characterized by repetitive or sustained teeth tightening and grinding or by the thrust of the mandible. It is not considered a movement disorder in otherwise healthy individuals (35). Sleep and awake bruxism, moreover, can be graded as follows: possible (based on a positive self-report only), probable (based on a positive clinical inspection with or without a positive self-report), and definite (based on a positive instrumental assessment) (35, 36). Evaluation of sleep bruxism and awake bruxism was carried out by a non-instrumental approach consisting of self-assessment, questionnaires, oral history, and clinical inspection. Clinical features of both awake and sleep bruxism included the presence of masticatory muscle hypertrophy, indentations on the tongue or lip, a Linea alba on the inner cheek, and presence of damaged dental hard tissues (e.g., worn or cracked teeth) in case of sleep bruxism (32, 35).

The data collected in Chieti Clinic were then compared with those that emerged in the Murcia Clinic, calculating the statistical value chi-square. As for the statistical analysis, the chi-square test was used to compare the two percentages obtained in the study and evaluate the statistical significance or verify whether the difference between the two values is due to chance. Everything was calculated at a 5% probability level, considering one degree of freedom and *n* = 411. To compare the pain intensity in masticatory muscles and the VAS values between groups, the Student's *t*-test for unpaired data was performed, setting the significance level at *p* < 0.05.

## Results

In this study, CG group and MG groups 56 and 43 patients, respectively, present TMJ disorders with a mean age of 10.3 ± 0.3 years. The χ^2^ chi-square test results revealed no differences between the two groups. The chi-square value (1, *n* = 411) is 1.057 and the *p*-value is 0.304, the chi-square with Yates correction is χ^2^ (1, *n* = 411) = 0.834 and the *p*-value is 0.361 (Table 1). Different TMD signs and symptoms in both groups were analyzed to investigate the relationship between the TMDs and the two groups (Table 2). There was a statistically significant higher prevalence of deviation of the mandible during opening (χ^2^ = 7.035; *p* = 0.008) and joint sounds (χ^2^ = 6.932; *p* = 0.008) in the MG group than in the CG group. For intra-articular disorders, disc displacement with reduction was found in 23.23% of patients considering both groups, with significant differences between the two groups (χ^2^ = 4.689; *p* = 0.030). Crepitus was found in only 2.02% of subjects considering both groups. No cases of disc displacement without reduction with/without limited opening were detected. Moreover, among patients in the CG group, awake bruxism was found in 85.71% of subjects, while it was 30.23% among MG ones (χ^2^ = 29.355; *p* = 0.000). The reduced opening movement was found in a total of seven subjects (range 32–36 mm; mean value 33.8 ± 1.6 mm) with no differences between the groups (χ^2^ = 0.183; *p* = 0.669). No differences were found about parafunctional habits (χ^2^ = 0.360; *p* = 0.548).

**Table 1:** Prevalence of TMJ disorders in the two group.

**Group**	**Age**	**TMD**		**No TMD**		**Tot**	**χ^2^**	***p*-Value**
		***n***	**%**	***n***	**%**			
CG group	11 ± 1.9	56	26.17 %	158	73.83%	214	1.057	0.304
MG group	10.9 ± 2.3	43	21.83%	154	78.17%	197		
CG + MG	10.9 ± 2.1	99	24.09%	312	75.91%	411	

CG group, Chieti group; MG group, Murcia group; TMD, patients with TMJ disorders; No TMD, patients without TMJ disorders; Tot, total number of patients.

χ^2^ test was used to verify the existence of statistical differences between the two groups.

**Table 2:** Prevalence of TMD signs and symptoms in each group.

	**CH group**		**MU group**		**Tot**		**χ^2^**	***p*-Value**
	***n***	**%**	***n***	**%**	***n***	**%**		
Sleep bruxism	16	28.57%	13	30.23%	29	29.29%	0.009	0.966
Awake bruxism	48*	85.71%*	13*	30.23%*	61	61.61%	29.355	0.000
Deviation during opening	12*	21.43%*	21*	48.84%*	33	33.33%	7.035	0.008
Joint sounds^a^	8*^a^	14.28%*	17*	39.53%*	25	25.25%	6.932	0.008
Click	8*^b^	14.28%*	15*^ac^	34.88%*	23	23.23%	4.689	0.030
Crepitus	0		2	4.65%	2	2.02%		
Disc displacement with reduction	8*	14.28%*	15*	34.88%*	23	23.23%	4.689	0.030
Reduced movements	5	8.93%	2	4.65%	7	7.07%	0.183	0.669
Parafunctional habits	8	14.28%	9	20.93%	17	17.17%	0.360	0.548
Local myalgia	34	60.71%	26	6.,46%	60	60.60%	0.033	0.855
Arthralgia	5*	8.93%*	12*	27.91%*	17	17.17%	4.897	0.027
Degenerative joint disease	0		2	4.65%	2	2.02%		

CG group, Chieti group with TMDs; MG group, Murcia group with TMDs.

χ^2^ test with Yates correction used to verify the existence of statistical differences between the two groups.

*Significantly higher than all the other signs and symptoms of temporomandibular disorders.

^a^Joint sounds with disc displacement with reduction.

^b^Four patients present opening click, four patients present both opening and closing click.

^c^Eleven patients present opening click, and four patients present both opening and closing click.

About Angle dental class, 37.37% of the subject with TMDs of both groups present molar Class I relationship with no differences between CG and MG groups. Significant differences between the two groups (χ^2^ = 3.951; *p* = 0.047) were observed in patients with Class III malocclusion as shown in Table 3.

**Table 3:** Prevalence of TMDs in association with different dental class.

	**CL I**		**CL II**		**CL III**		**D CL**		**χ^2^**	***p*-Value**
	***n***	**%**	***n***	**%**	***n***	**%**	***n***	**%**		
CG group	20	35.71%	16	28.57%	12*	21.43%*	8	14.28%	4.547	0.208
MG group	17	39.53%	13	30.23%	3*	6.98%*	10	23.25%		
CG + MG	37	37.37%	29	29.29%	15	15.15%	18	18.18%		

CG group, Chieti group with TMDs; MG group, Murcia group with TMDs; CL I, Class I; CL II, Class II; CL III, Class III; D CL, different class on the two sides.

χ^2^ test was used to verify the existence of statistical differences between the two groups.

*Significantly higher than the other entire dental class groups: χ^2^ = 3.951 p = 0.047.

Moreover, the intraoral examination revealed no differences between CG and MG subjects regarding overbite, posterior, and anterior crossbite, while significant differences among the two groups were observed in deep bite (range4–7 mm; mean value 5.4± 1.5 mm), (χ^2^ = 8.619; *p* = 0.003) and an increased overjet (range 3–11 mm; mean value 4.7± 1.8 mm), (χ^2^ = 7.588; *p* = 0.006; Table 4).

**Table 4:** Prevalence of TMDs in association with occlusal variables.

	**DB**		**OB**		**PCB**		**ACB**		**Tot CB**		↑**OJ**		↓**OJ**	
	***n***	**%**	***n***	**%**	***n***	**%**	***n***	**%**	***n***	**%**	***n***	**%**	***n***	**%**
CG group	32*^a^	57.14%*	8	14.28%	16	28.57%	8	14.28%	24	42.86%	16**^b^	28.57%**	8	14.28%
MG group	11*^a^	25.58%*	4	9.30%	7	16.28%	4	9.30%	11	25.58%	25**^b^	58.14%**	4	9.30%
CG + MG	43^a^	43.43%	12	12.12%	23	23.23%	12	12.12%	35	35.35%	41^b^	41.41%	12	12.12%

CG group, Chieti group with TMDs; MG group, Murcia group with TMDs; DB, deep bite; OB, open bite, PCB; posterior crossbite; ACB, anterior crossbite; Tot CB, total crossbite; ↑OJ, increased overjet; ↓OJ= inverted overjet.

χ^2^ test and χ^2^ test with Yates correction were used to verify the existence of statistical differences between the two groups.

*Significantly higher than all the other groups: χ^2^ = 8.619 p = 0.003.

**Significantly higher than all the other groups: χ^2^ = 7.588 p = 0.006.

^a^(Range 4–7 mm).

^b^(Range 3–11 mm).

About TMD pain-related, 60.60% of patients (*n* = 60) present local myalgia with no difference between the two groups and 17.17 % of patients (*n* = 17) present arthralgia with statistically differences between the two groups (χ^2^ = 4.897; *p* = 0.027) as shown in Table 2. There was no difference between the two groups regarding the prevalence of TMD pain-related in masticatory muscles except in temporalis tendon palpation (χ^2^ = 4.632; *p* = 0.031; Table 5). No statistical differences between the two groups were found in the intensity of pain in masticatory muscles, as shown in Table 6. The VAS values are shown in Table 7. Significant differences between the two groups were observed among patients aged 10 years (*p* = 0.020), 11 years (*p* = 0.004), and 13 years (*p* = 0.003). Considering both groups, higher VAS values were observed in patients aged 11–15 years (range 5.33–8.0; mean 6.04 ± 2.14).

**Table 5:** Prevalence of TMD pain-related in masticatory muscles.

	**M**		**T**		**Pt**		**Tt**		**D**	
	***n***	**%**	***n***	**%**	***n***	**%**	***n***	**%**	***n***	**%**
CG group *n* = 56	13	23.21%	6	10.71%	17	30.36%	16*	28.57%*	3	5.35%
MG group *n* = 43	8	18.60%	4	9.30%	12	27.91%	4*	9.30%*	4	9.30%
CG + MG *n* = 99	21	21.21%	10	10.10%	29	29.29%	20	20.20%	7	7.07%

CH group, Chieti group with TMDs; MU group; Murcia group with TMDs; Pt, lateral and medial pterygoids; M, masseter; T, temporal; Tt, temporalis tendon; D, digastric anterior belly.

χ^2^ test and χ^2^ test with Yates correction were used to verify the existence of statistical differences between the two groups.

*Significantly higher than all the other muscle palpation groups: χ^2^ = 4.470 p = 0.034.

**Table 6:** Intensity of pain in masticatory muscles (score 0–3) in the two group.

	**M**		**T**		**Pt**		**Tt**		**D**	
	**Mean ±SD**	***p*-Value**	**Mean ±SD**	***p*-Value**	**Mean ±SD**	***p*-Value**	**Mean ±SD**	***p*-Value**	**Mean ±SD**	***p*-Value**
CG group	1.44 ± 0.70	0.249	1.50 ± 0.70	0.418	1.67 ± 0.75	0.506	1.50 ± 0.69	0.060	2 ± 1.15	1
MG group	1.79 ± 0.89		1.86 ± 0.90		1.81 ± 0.81		2.14 ± 0.89		2 ± 1	
CG + MG	1.60 ± 0.81		1.66 ± 0.82		1.72 ± 0.77		1.74 ± 0.81		2 ± 1	

CG, Chieti group; MG, Murcia group; SD, standard deviation; M, masseter; T, temporal; Pt, lateral and medial pterygoids; Tt, temporalis tendon; D, digastric anterior belly.

t-test used to verify the existence of statistical differences between the two groups.

**Table 7:** VAS values in the two groups.

**Age (7–15 y.o.)**	**CG VAS**		**MG VAS**		**CG + MG**	***p*-Value**
	***n***	**Mean ±SD**	***n***	**Mean ±SD**	**Mean ±SD**	
7				
8	6	3.67 ± 0.52	5	3.60 ± 0.89	3.63 ± 0.67	0.880
9	3	4.33 ± 1.15	2	1.50 ± 0.70	3.20 ± 1.78	0.056
10	2	6.50 ± 0.70	4	2.50 ± 1.73	3.83 ± 2.48	0.020*
11	12	4.75 ± 2.60	9	7.67 ± 1.58	6 ± 2.63	0.004*
12	8	4.75 ± 2.43	4	6.50 ± 1	5.33 ± 2.19	0.102
13	6	7.17 ± 1.47	5	4.40 ± 0.52	5.90 ± 1.81	0.003*
14			9	6.77 ± 0.66	6.77 ± 0.66	
15	2	8 ± 1.41		8 ± 1.41	

CG, Chieti group; MG, Murcia group; VAS, visual analog scale (score 0–10); SD; standard deviation.

t-test for unpaired data used to verify the existence of statistical differences between the two groups.

*Significantly higher than all other groups.

## Discussion

To improve the statistical analysis, two samples were analyzed from two demographically, culturally, and economically similar countries, namely, Italy and Spain. The prevalence of TMD symptoms was 26.17% in the Chieti group and 21.83% in the Murcia group, with no significant differences between the two groups. The majority of TMD was 24.09%, considering both groups. The prevalence of TMD is not well known yet, and according to Pahkala and Laine (37), during childhood, there is the transition from deciduous to permanent dentition with the modification of the craniofacial complex and adaptive physiological changes in TMJs, and it is essential to investigate the signs and symptoms of TMD in children and adolescents through epidemiological studies. Malocclusion should not be excluded entirely as an etiologic factor of TMD, although it is still necessary to investigate how much it contributes to its onset (38). Some clinicians suggest that occlusal conditions such as deep bites, crossbites, and double bites are predisposing factors, and other factors such as trauma, emotional stress, bruxism, and some systemic conditions may also be responsible for developing a TMJ disorder. The absence of bilateral canine guidance on a lateral excursion and particularly Angle Class II malocclusion was considered essential risk indicators for the development of TMD (39, 40). Open bite, deep bite, and posterior crossbite seemed to be the most important associated with TMD (41, 42). In contrast, two studies (43, 44). concluded no relationship between dental classification and TMD. Pullinger et al. (45) evaluated 11 occlusal variables comparing asymptomatic controls vs. five TMDs groups. They concluded that occlusion could not be considered the primary factor in defining TMDs. Many occlusal parameters contribute to the onset of TMDs in a minor way than believed.

In addition, some occlusal features have been considered a consequence rather than etiological factors for the disorder, such as the anterior open bite in patients with osteoarthrosis. In Akeel and Al-Jasser (46), no significant association was found between IOTN (Index of Orthodontic Treatment Need) and TMD signs and symptoms. Malocclusion could not be considered a primary etiologic factor for TMD within the age range studied. Runge et al. (47) concluded that a wider interincisal angle and an increased overbite were associated with joint noises. Sadowsky et al. (48), instead, found no significant connection between joint noise and functional occlusion. In Aboalnaga et al. (38), the total TMD sample showed an Angle Class I molar relation as the highest percentage (56.7%) than Classes II division 1 (20.7%), division 2 (8.7%), and Class III (12.7%). In this study, the prevalence of TMD signs and symptoms, considering both groups, was observed in 37.4% of subjects with dental Class I and 29.3% of subjects with dental Class II. TMD signs and symptoms were observed in 15.5% of subjects with dental Class III as shown in Table 3.

A child's emotional status may influence the risk of developing signals of TMD, as reported by several studies (48–50). High stress and anxiety levels can result in a constant clenching of the teeth with consequent alteration of the local circulation of the muscles, and this leads to an increase of the lactic and pyruvic acids with resultant stimulation of pain receptors (51).

The association between anxiety and malocclusion and the TMD prevalence was studied in the cross-sectional study conducted by de Paiva Bertoli et al. (52). Adolescents with high anxiety had a prevalence of TMD symptoms 4.06 times greater, while adolescents with moderate anxiety levels had a prevalence of TMD symptoms 1.94 times greater, regardless of gender.

Karibe et al. (53) found a significant association between head-forward posture and TMD in adolescent subjects. However, Olivo et al. (54) concluded that head-forward posture was not a significant risk factor for TMD symptoms, and the relationship between advanced head position and TMDs remains inconclusive. Parafunctional habits overloading the masticatory system could play an etiological role in developing TMDs (55). Awake bruxism and night grinding of teeth, for example, have been reported to be associated with TMDs in children and adolescents (56). A case series study conducted by Festa et al. (57), which used magnetic resonance imaging (MRI) of TMJ and functional nuclear magnetic resonance of the brain, reported the correlation between awake bruxism and TMD. The present study found that sleep bruxism was present in about 29% (with no significant difference between the two groups) and awake bruxism in about 61% of total subjects. The presence of joint sounds was found in about 25% of subjects according to the study by Bonjardim et al. (58) that detected TMJ sounds as the most prevalent symptoms (26.72%). The prevalence of clicking with disc displacement with reduction was found in about 23% of subjects, as reported in Table 2, higher than that observed in Bertoli et al. (27) (8%) study. This difference is probably because TMD signs and symptoms, especially clicking sounds, in developmental age, are often occasional and generally increase with age (59). In the present study, crepitus was detected in about 2% of subjects; according to other authors, it is uncommon among children and adolescents (2–4, 27, 60). In addition, while several studies have reported a correlation between oral parafunctional habits and TMD symptoms in children and adolescents (61, 62), others have disputed these correlations (63, 64), Thilander et al. (65) and Bilgiç and Gelgor (66) found a significant association between Class III and TMD. Bilgiç and Gelcor (66) found that headache was the only TMD symptom reported by the children.

Furthermore, 25% of the subjects reported more than one clinical sign, most mild. The prevalence increased during the developmental age. Girls were more affected than boys, and TMD was associated with a posterior crossbite, anterior open bite, Angle Class II and III malocclusions, and increased overjet (66).

An altered occlusion can cause disorders in oral function and problems of a psychosocial nature due to the dentofacial aesthetic compromise, and a high prevalence of malocclusions has been reported in children and adolescents, ranging from 39 to 93% (67). The findings in the current study regarding malocclusions are illustrated in Table 4. Deep bite, increased overjet, and anterior/posterior crossbite were found in about 43, 41, and 35% of subjects, respectively Local myalgia was observed in about 60% of subjects, while arthralgia in about 17% of subjects, in agreement with other studies that reported as the most prevalent diagnosis myofascial pain (27, 68). The lateral and medial pterygoids and the masseter were the most painful observed muscles.

Perrotta et al. (69) found a significant association between TMD pain and negative overbite, unilateral, and bilateral crossbite. The study of Tecco et al. (70) emerges that the TMD signs and symptoms were 1.6 times more frequent in subjects with Class II/first division than subjects in Class I, as well as joint noises (2.75 times more frequent). Women had a higher prevalence (1.96 times) for myalgia and were statistically significant than men.

Finally, the study by Tecco et al. (23) demonstrated a higher prevalence of myofascial pain among subjects aged between 12 and 15 years compared to those aged 5–11 years (about 14% of prevalence vs. 5% of prevalence) and also a higher prevalence in the women (10% vs. the 5% observed among men). In addition, TMD signs and symptoms and reduced functional movements were found more frequently in subjects with unilateral posterior crossbite (60%) than in subjects with an anterior or posterior bilateral crossbite.

Rinchuse and McMinn (71), comparing various systematic reviews, found few associations between malocclusion or functional occlusion and TMD signs and symptoms. The only correlations were between crowded posterior teeth, subjective dysfunction symptoms, and evident abrasions and clinical dysfunctions. An increase in pain conditions, such as headache, abdominal pain, and musculoskeletal pain, has been associated with puberty, a period characterized by hormonal, physical, and psychosocial changes that may influence temporomandibular disorders' onset and maintenance. In the systematic review of Song et al. (72), the association between TMD and pubertal development was studied, and the prevalence of temporomandibular pain (of the masticatory muscles or the TMJ) increases with the advancement of pubertal development; in fact, it affected about 4% in pre-pubertal subjects and 14% in subjects who had completed pubertal development (73). There is substantial literature to support the role of genetic, psychosocial factors, and muscle-related overload; however, the association between various features of dental occlusion and TMD is lacking (14, 16, 74–76). The current cross-sectional study found an association between occlusal variables such as deep bite in about 43% of subjects or increased overjet in about 41% of subjects, but, according to other studies (14, 16), the presence of any association does not imply causation. This study found the presence of posterior crossbite in about 23% of subjects. It could not conclude that it is causative of TMD disorders according to Michelotti et al. (77); probably, it could be a consequence of the individual skeletal morphology (14).

This study's research limit is the absence of a control group to strongly verify the association between malocclusion and TMDs as specific etiological factors. Furthermore, another limit is the no use of polysomnography (PSG) to diagnose sleep/awake bruxism. The aim for future studies is to increase the sample size, namely, a control group and the use of PSG, on which inclusion will be relevant.

## Conclusion

The results in the current study indicate that the prevalence of TMDs is 26.17% in patients with Chieti and 21.83% in patients with Murcia, considering joint sounds, the presence of sleep and awake bruxism, and opening deviation as pathognomonic signs.

This study observed a significant association between TMDs and deep bite (43.43%), increased overjet (41.41%), and posterior crossbite (23.23%), considering the occlusal interferences and between the Angle molar Class I (37.37%), considering malocclusions.

Finally, it is possible to conclude that there is also a mandibular deviation in the opening, joint sound, tooth grinding, and bruxism.

## Data availability statement

The raw data supporting the conclusions of this article will be made available by the authors, without undue reservation.

## Ethics statement

The study was conducted in the Oral Sciences Department of the University of Chieti G. D'Annunzio. The study protocol was designed in accordance with the European Union Good Practice Rules and with the Helsinki Declaration. Ethical approval (number 23) was obtained from the hospital's Independent Ethics Committee of Chieti. Each patient provided written informed consent to participate in this study. The patients/participants provided their written informed consent to participate in this study.

## Author contributions

FF and MM designed the study. FF, MM, AS, and GM selected the sample and analyzed the data. MM wrote the manuscript and revised it before submission. All authors read and approved the final manuscript.

## Conflict of interest

The authors declare that the research was conducted in the absence of any commercial or financial relationships that could be construed as a potential conflict of interest.

## Publisher's note

All claims expressed in this article are solely those of the authors and do not necessarily represent those of their affiliated organizations, or those of the publisher, the editors and the reviewers. Any product that may be evaluated in this article, or claim that may be made by its manufacturer, is not guaranteed or endorsed by the publisher.
